# Discovering gene functional relationships using FAUN (Feature Annotation Using Nonnegative matrix factorization)

**DOI:** 10.1186/1471-2105-11-S6-S14

**Published:** 2010-10-07

**Authors:** Elina Tjioe, Michael W Berry, Ramin Homayouni

**Affiliations:** 1 Department of Electrical Engineering and Computer Science and Graduate School of Genome Science and Techonology, University of Tennessee, Knoxville, TN 37996, USA; 2 Department of Biology, Bioinformatics Program, University of Memphis, Memphis, TN 38152, USA

## Abstract

**Background:**

Searching the enormous amount of information available in biomedical literature to extract novel functional relationships among genes remains a challenge in the field of bioinformatics. While numerous (software) tools have been developed to extract and identify gene relationships from biological databases, few effectively deal with extracting new (or implied) gene relationships, a process which is useful in interpretation of discovery-oriented genome-wide experiments.

**Results:**

In this study, we develop a Web-based bioinformatics software environment called FAUN or Feature Annotation Using Nonnegative matrix factorization (NMF) to facilitate both the discovery and classification of functional relationships among genes. Both the computational complexity and parameterization of NMF for processing gene sets are discussed. FAUN is tested on three manually constructed gene document collections. Its utility and performance as a knowledge discovery tool is demonstrated using a set of genes associated with Autism.

**Conclusions:**

FAUN not only assists researchers to use biomedical literature efficiently, but also provides utilities for knowledge discovery. This Web-based software environment may be useful for the validation and analysis of functional associations in gene subsets identified by high-throughput experiments.

## Background

The MEDLINE 2010 literature database at NIH contains over 19 million records and is growing at an exponential rate [1]. With such rapid growth of the biomedical literature and the breakdown of disciplinary boundaries, it can be overwhelming to manually track all new relevant discoveries, even on specialized topics. Moreover, the recent advances in genomic and proteomic technologies have added an abundance of genomic information into biomedical knowledge, which makes the situation even more complicated. One main difficulty in understanding high-throughput genomic data is to determine the functional relationships between genes.

By design, high throughput experimental approaches are expected to yield new discoveries. For example, gene expression profiling by DNA microarray technology can identify hundreds of genes whose expression is co-regulated with experimental treatments. The researcher is expected to reduce this list to functional pathways and mechanisms that can be further investigated experimentally. While some of the differentially expressed genes may be known to functionally interact, it is expected that many interactions are implied and weakly supported in the literature. Therefore, there is a growing need to develop new text-mining tools to assist researchers in discovering hidden or implicit functional information about genes directly from biomedical literature. Surveys of online tools for literature-based discovery in the life sciences are available [2,3], and a more recent article by Roos et al. [4] describes the integration of Semantic Web technologies with text extraction and mining for hypothesis generation within a workflow environment.

### Previous work

Numerous data mining tools have been proposed for bioinformatics research (see reviews in [5-9]). One of the major steps in text mining is information retrieval (IR) [6] which consists of three basic types of models: set-theoretic (Boolean), probabilistic, and algebraic (vector space). Documents in each case are retrieved based on Boolean logic, probability of relevance to the query, and the degree of similarity to the query, respectively.

Some of the current software tools utilize functional gene annotations provided in public databases, such as Gene Ontology (GO) [10], Medical Subject Heading (MeSH) index [11], and KEGG [12]. For example, GoPubMed [13], a thesaurus-driven system, classifies abstracts based on GO, HAPI [14] identifies gene relationships based on co-occurrence of MeSH index terms in representative MEDLINE citations, and EASE [15] identifies gene relationships using the gene function classifications in GO. These co-occurrence based methods can be highly error prone. Ontology definitions help provide insights into biological processes, molecular functions and cellular compartments of individual genes. However, they are often incomplete and lack information related to associated phenotypes [16]. In addition, Kostoff et al. [17] found a significant amount of conceptual information present in MEDLINE abstracts missing from the manually indexed MeSH terms. Moreover, indexing in MEDLINE can be inconsistent because of assignments by different human-indexers [18].

Several alternative approaches that use Medline-derived relationships to functionally group related genes have been reported [19]. Alako et al. [20] have developed CoPub Mapper which identifies shared terms that co-occurred with gene names in MEDLINE abstracts. PubGene [21] developed by Jenssen et al. constructs gene relationship networks based on co-occurence of gene symbols in MEDLINE abstracts. Because of the inconsistency issues in gene symbol usage in MEDLINE, PubGene has low recall (ratio of relevant documents retrieved to the total number of relevant documents). It identifies only 50% of the known gene relationships on average. In addition to the official gene symbol, each gene typically has several names or aliases. In IR, these problems are referred to as synonymy (multiple words having the same meaning) and polysemy (words having multiple meanings). Several methods have been proposed to solve these ambiguity issues in gene or protein names [22-24].

The concept of literature based discovery was introduced by Don Swanson in 1986 [25] and has since been developed and applied to many different areas of research [26,27]. Many online literature-based discovery tools have been developed, some of which have resulted in documented discoveries [2]. Chilibot [28], Textpresso [29], and PreBIND [30] are examples of tools that are specifically geared toward genomic and proteomic applications [31]. Chilibot is a system with a special focus on the extraction of relationships between genes, proteins and other information. Textpresso is an information-retrieval tool for biological entities that was originally designed for WormBase and later applied to other model organisms. Finally, PreBIND provides utilities in the extraction of protein-protein interactions. One drawback of many of these existing tools is that they are not amenable to analysis of high throughput genomic experiments which result in hundreds or even thousands of genes that must be further analyzed.

We previously developed a text-mining tool called Semantic Gene Organizer(SGO) [32], which implements Latent Semantic Indexing (LSI) to extract functional relationships among genes from MEDLINE abstracts. Homayouni et al. [33] demonstrated that SGO extracted both explicit (direct) and implicit (indirect) gene relationships based on keyword queries, as well as gene-abstract queries, from the biomedical literature with better accuracy than term co-occurrence methods. The underlying SVD factorization technique decomposes the original term-by-document nonnegative matrix into a new set of factor matrices containing positive and negative values. These matrix factors can be used to represent both terms and documents in a low-dimensional subspace. Unfortunately, the interpretation of the LSI factors is non-intuitive and difficult to interpret due to the negative factor components. The main limitation of LSI is that while it is robust in identifying* what* genes are related, it has difficulty in answering* why* they are related.

To address this issue, a new method called* nonnegative matrix factorization* (NMF) has been developed for genomic applications. Unlike SVD, NMF produces decompositions that can be readily interpreted.

### NMF background

Lee and Seung [34] were among the first researchers to introduce the nonnegative matrix factorization (NMF) problem. They demonstrated the application of NMF in text mining and image analysis. NMF decomposes and preserves the nonnegativity of the original data matrix. The low-rank factors produced by NMF can be interpreted as parts of the data. Recently, NMF has been widely used in the bioinformatics field, including the analysis of gene expression data, sequence analysis, gene tree labeling, functional characterization of gene lists and text mining [35-41]. Chagoyen et al. have shown the usefulness of NMF methods in extracting the semantic features in biomedical literature [36]. Pascual-Montano et al. [40] developed an analytical tool called bio-NMF for simultaneous clustering of genes and samples. It requires (on input) a data matrix (e.g., term-by-doc matrix) and outputs the corresponding matrix factors. Even though the tool is robust and flexible, its use by biologists might not be obvious. Therefore, an intuitive interface that allows the biologist to use literature-based NMF methods for determining functional relationships among genes is still needed.

In this study, we develop a Web-based bioinformatics software environment called Feature Annotation Using Nonnegative matrix factorization (FAUN) to facilitate both knowledge discovery and classification of functional relationships among genes. The ability to facilitate knowledge discovery makes FAUN very attractive to genomic scientists. Thus, one of the main design goals of FAUN is to be biologically user-friendly. Providing a list of genes with gene identifiers such as gene IDs or gene names, FAUN constructs a gene-list-specific document collection from the biomedical literature. NMF can be used to exploit the nonnegativity of term-by-gene document data, and can extract the interpretable features of text which might represent usage patterns of words that are common across vastly different gene documents. NMF methods are iterative in nature so that the problem involves computational issues such as: proper initialization, rank estimation (i.e., subspace dimension), stopping criteria, and convergence. To address these issues, many variations of NMF with different parameter choices have been proposed [38,42,43]. While developing FAUN, we try to understand how the NMF model can be adapted or improved for gene datasets that will not only yield good mathematical approximations, but also provide valuable biological information.

## Methods

A simple demonstration of the FAUN bioinformatics software environment (using an anonymous login) is available to the public at https://grits.eecs.utk.edu/faun; accounts can be created upon request and an* upload* feature for creating NMF models of user-supplied genelists is available.

### Extracting concept features

**Figure 1:**
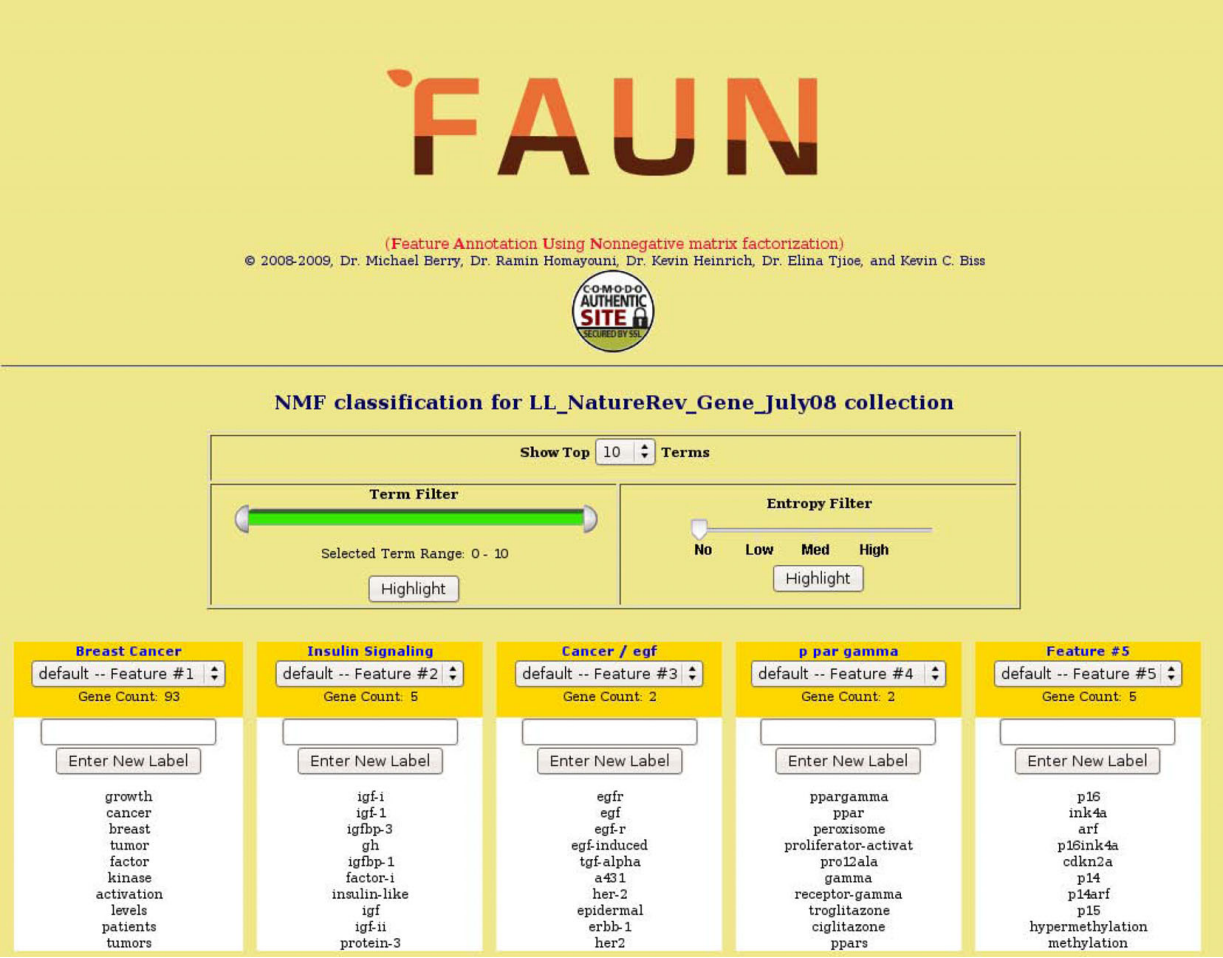
**Five FAUN-generated features for the NatRev collection (110 genes) along with their top (highest intensity) terms.** Available options such as* show-terms* and* term and entropy filtering* are shown as pull-down menus or slider bars.

Due to the nonnegativity constraints of the NMF model, interpretable features can be extracted from the columns in the term-by-feature (*W*) matrix. Since the matrix is sparse, each column (feature) is represented by a small subset of terms, which forms a certain term usage pattern. This pattern will help FAUN users in determining the concept or meaning of the feature. For example, a feature containing the terms* mosquito, Plasmodium, blood,* and* quinine* might describe the disease malaria. Once the user recognizes a specific feature, based on its dominant terms, the feature can be annotated into something more meaningful (e.g., breast cancer) than the default label (e.g., Feature 1) for future reference. The screenshot of some annotated features and their top associated terms for the NatRev gene document collection (dataset 3) are shown in Figure 1. The entropy filter slider bar allows the user to get information on how the term is used consistently throughout the whole collection. If a term occurs in all documents the same way, it will have a low entropy weight and is probably not a very good concept discriminator. High entropy words tend to have specific usage patterns and are hopefully more meaningful. This entropy filter option might help the user to focus on more important features.

A typical usage scenario for FAUN concept features is shown in Figure 1. Once a document collection is built, three NMF models are generated with NMF ranks *k* = 10, 15, and 20, for low, medium, and high resolutions by default. Even higher resolutions are certainly possible, and the screenshot in Figure 1 is taken from a NMF model with 30 features (only the first 5 features are shown). The user can then look through the top terms in each feature and supply (if possible) an appropriate label. For example, Feature 2 in Figure 1 could readily be labeled (or annotated) as a descriptor of insulin signaling.

If the user is interested in further exploring the* Insulin Signaling* feature, he/she can then click on the feature to show all the genes in the collection that FAUN suggested to be highly associated with the feature. A description of how FAUN identifies the genes associated with each feature is provided in the next section.

### Gene identification

For each feature, genes that are highly associated with it can be extracted from the feature-by-gene (*H*) matrix. A gene can be described by more than one feature. The association strength (feature weight) between gene and feature is determined by the appropriate element in the matrix. Genes that share one or more of the same features might be functionally related to one another.

**Figure 2:**
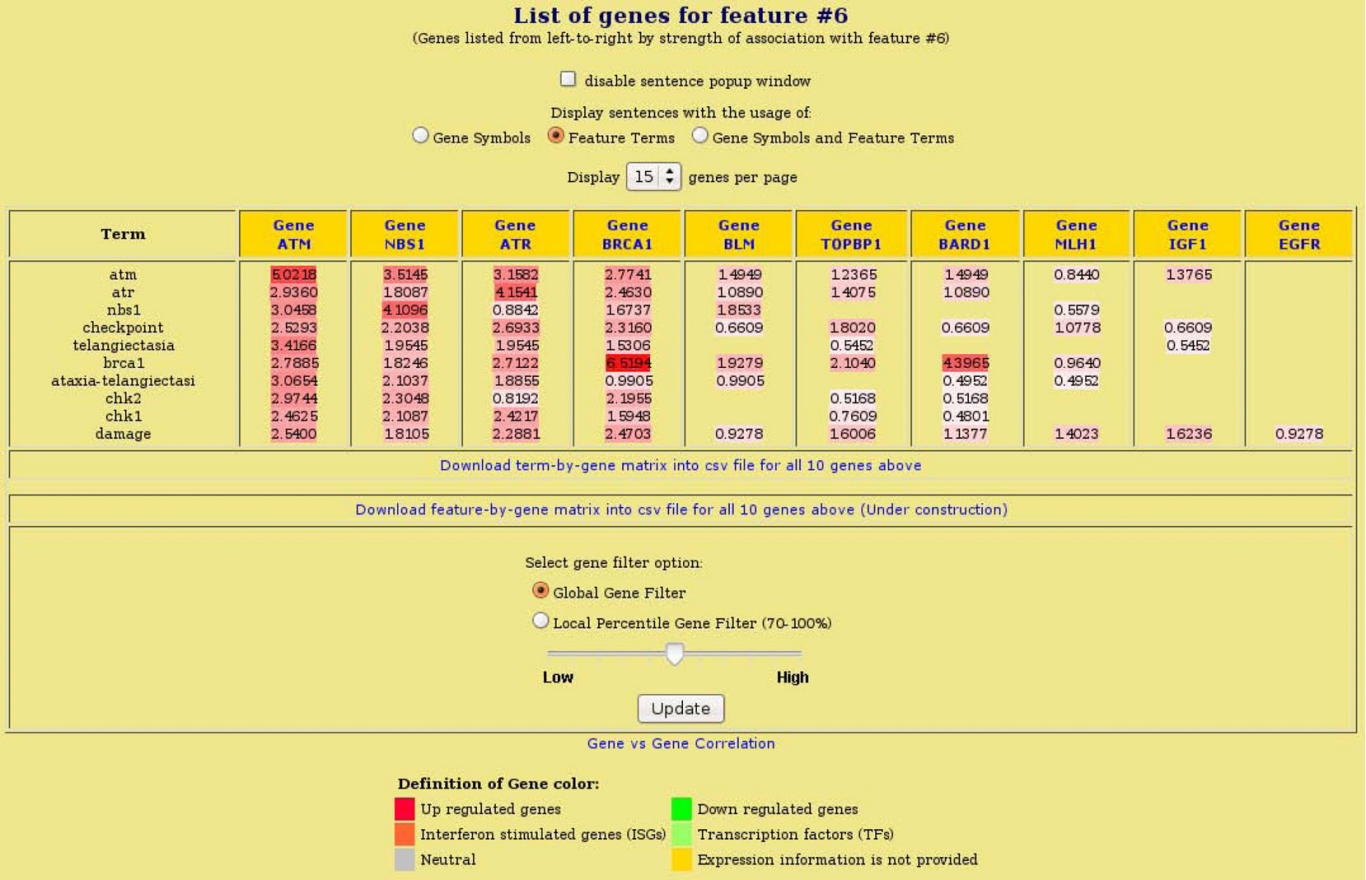
**Display of the dominant terms of Feature 6 (DNA damage/ATM) from the higher resolution (rank-30) NMF model of the NatRev collection as they occur in genes highly associated with that feature.** The* gene filtering* option uses thresholding on components of the *H* matrix factor in the NMF to vary the number of genes displayed. The* display sentences* option allows the user to view a ranked list of sentences (based on term frequency) from any particular gene (document).

The genes in the gene document collection can also be color-coded based on their expression change in the microarray experiments: red is up-regulated and green is down-regulated. The user can see not only which genes belong to what features, but also see if a particular feature contains predominantly up- or down-regulated genes in the userâspecific experiments. The feature is color-coded based on the predominant genes in that feature which are up- or down-regulated. If gene expression information is not provided, the gene and its feature are color-coded with yellow as shown in Figures 1 and 2. The screenshots of* genes* that are highly associated with Feature 6 and their top associated* terms* for the NatRev collection, along with some options, are shown in Figure 2.

Genes are listed from left-to-right by strength of association with the selected feature. The log-entropy weight of the terms in each gene is color-coded for visual analysis, with more red for a higher weight. The number of genes to be displayed is set to 15 and can be changed using the display-genes drop-down menu. All genes, above the set feature weight threshold, with their terms and term weights can be downloaded in csv format for further analysis. There might not be a single optimal threshold value that works the best for every case. FAUN provides global and local gene filter options to let users try different thresholds. The gene filter option allows users to filter the genes associated with each feature globally, across all gene documents above a certain threshold, or locally, within each gene document above the 70th local percentile. The* medium* global gene filter setting (i.e., feature weight = 1.0) is the default.

To see how the feature terms and/or gene symbols are used in the original gene document article, sentences using the terms and/or the gene symbols can be viewed. The sentences are ranked based on term frequency. The ranked sentences are displayed in the popup window by clicking on the gene symbol at the head of the column. This popup window also serves as the quick summary page for the gene and provides a link to the Entrez Gene page for more information about that gene.

At this point, the FAUN user might have some ideas about what kinds of features are present in the gene document collection, and some familiarity with the genes that are associated with certain features. Genes belonging to the same feature might suggest that they are functionally related based on the literature. Such hypotheses may well lead to new discoveries in gene characterization. Namely, genes represented by the same feature may function in the same pathway.

To explore even further why certain subsets of genes are related, and how strongly they are related, the user can click on the* gene vs gene correlation* link shown at the bottom of the screenshot in Figure 2.

### Gene exploration

FAUN’s capability to identify subsets of genes which are related is described above. To see how strongly genes in the user-selected feature (*i*) cross-correlate, the correlation of gene *x* and gene *y* for *n* selected features is estimated using the Pearson correlation coefficient (*r*) which is defined by

	(1)

**Figure 3:**
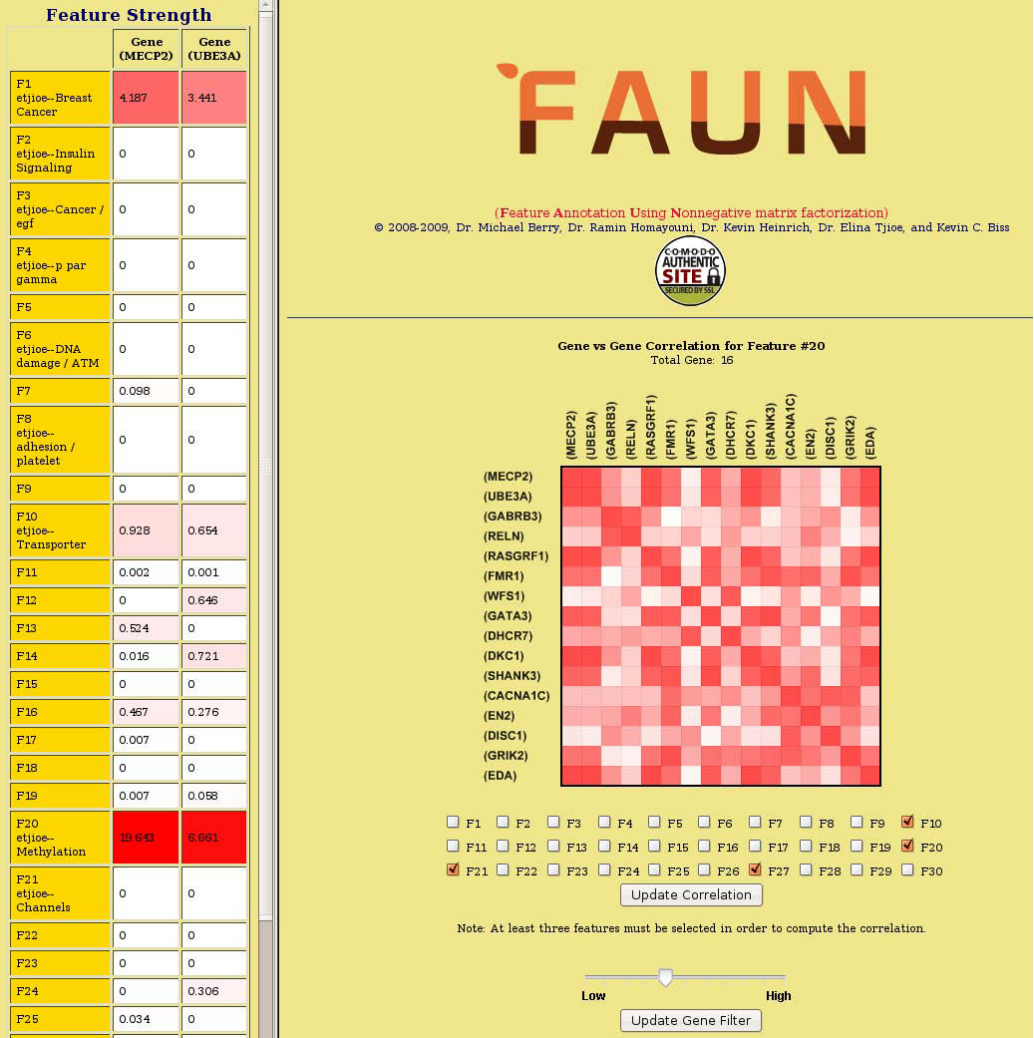
**An illustration of the gene-to-gene correlation FAUN option based on the Pearson correlation of gene features.** The rightmost window shows the correlation between genes highly associated with the user-selected features 10, 20, and 27; the leftmost window shows the feature strength (and manually annotated labels) for the genes from the user-selected correlation cell.

The Pearson correlation matrix for all the genes is then generated. The correlation is color-coded for visual analysis, with more red for a stronger correlation. An example of the correlation matrix for all the genes in Feature 20 (*Methylation*) of the NatRev collection is shown in Figure 3. Users can view the correlation of any pair of genes and with respect to any combinations of features with a minimum of 3 features selected. By default, the user-selected feature along with its left and right neighboring features are used to compute the Pearson correlation.

Another important capability of FAUN is to potentially explain why a subset of genes might be related. Two pairs of genes which associate with different contributions of features might have an overall similar degree of correlation. In FAUN, the users can view exactly which contributions of (annotated and/or un-annotated features) are involved by clicking on the correlation cell shown in Figure 3. The selected cell in the figure shows a strong correlation, indicated by the red cell color, between gene **MECP2** and gene **UBE3A**, mainly due to Features 1 (*Breast Cancer*) and 20 (*Methylation*), suggested by the feature strengths at the left side.

### Gene classification

A new gene document added to a gene document collection can be analyzed (for the presence of annotated features) without having to update the NMF model for the collection. The FAUN classifier can accept a stream of new documents and determine their features based on the presence of terms in the previously-annotated features. It is be possible to automatically retrieve newly published articles and run the FAUN classifier to determine if they are related to any of the interest features in the studied gene collection without having to continually update the NMF model. Of course, periodic updating of the NMF model to reflect changes in literature may be needed.

### Software design

FAUN consists of a computational core and Web-based user interface. The computational core consists of programs that build the gene document collection, parse the collection, build an NMF model from the collection, and classify new documents based on the NMF model. These programs will be described in more detail in the following sections. The primary design goal of the user interface was to make the analysis of NMF accessible to biologists.

FAUN users can have one or more separate accounts to perform independent analysis of gene datasets. The sessions persist between logins so users can easily resume their work after interruption. The users can take advantage of various interactive components such as the gene-to-gene correlation matrix, sentence display, filters and dynamic generation of results. FAUN utilizes a combination of technologies: PHP, Javascript, Flash, and C++. PHP is used to communicate between the computational core and the Web user interface. It is also used for generating HTML pages. Javascript is used for client-side scripting and creating graphical user interface (GUI) elements. The gene-to-gene correlation matrix is generated using a PHP/SWF chart tool [44]. This tool is used for simple and flexible chart generation, and quality of Flash graphics. PHP scripts are used to generate chart data, and then the data is passed to the PHP/SWF chart tool to generate Flash charts and graphics. C++ was used to write the computational core.

### Gene document collection

All genes in a given gene list are used to compile titles and abstracts in Entrez Gene [45]. Currently, to avoid polysemy and synonymy issues, there are still human interventions in the document compilation process, such that abstracts are not specific to a particular gene name or alias. Titles and abstracts for a specific gene are concatenated to prepare a gene document.

The collection of gene documents is parsed into terms using the current C++ version of General Text Parser (GTP) [46]. Terms in the document collection that are common and non-distinguishing are discarded using a stoplist (see ftp://ftp.cs.cornell.edu/pub/smart/English.stop for a sample stoplist). In addition, terms that occur less than twice locally in the gene document or globally in the entire document collection are ignored and not considered as dictionary terms. Hyphens and underscores are considered as valid characters. All other punctuation and capitalization are ignored.

A term-by-gene document matrix is then constructed where the entries of the matrix are the nonnegative weighted frequencies for each term. These term weights, computed using a log-entropy weighting scheme [47], are used to describe the relative importance of term *i* for the corresponding (gene) document *j*. That is, the term-by-gene document matrix is defined as

*A* = [*w_ij_*], where *w_ij_* = *l_ij_* × *g_i_* .

The local component *l_ij_* and the global component *g_i_* can be computed as

	(2)

where ƒ*_ij_* is the frequency of term *i* in document *j*, *p_ij_* is the probability of the term *i* occurring in document *j* and *n* is the number of gene documents in the collection. This log-entropy weighting pair, which has performed well in several LSI-based retrieval experiments, decreases the effect of term spamming while giving distinguishing terms higher weight.

To summarize, a document collection can be expressed as an *m* × *n* matrix *A*, where *m* is the number of terms in the dictionary and *n* is the number of documents in the collection. Once, the nonnegative matrix *A* has been created, nonnegative matrix factorizaton (NMF) is performed.

### Nonnegative matrix factorization

NMF is a matrix factorization algorithm to best approximate the matrix *A* by finding reduced-rank nonnegative factors *W* and *H* such that *A* ≈* WH*. The sparse matrix *W* is commonly referred to as the *feature matrix* containing* feature (column) vectors* representing certain usage patterns of prominent weighted terms, while *H* is referred to as the* coefficient matrix* since its columns describe how each document spans each feature and to what degree.

In general, the nonnegative matrix factorization (NMF) problem can be stated as follows: Given a nonnegative matrix *A* ∊ *R^ m^*^×^*^ n^*, and an integer *k* such that 0 <*k* ≤ min(*m*, *n*), find two nonnegative matrices *W* ∊ *R^ m^*^ ×^*^k^* and *H* ∊ *R^ k^*^ ×^*^ n^* that minimize the cost function

	(3)

This cost function, half of the squared Frobenius norm of the residual error, equals 0 if and only if *A* =* WH*. The minimization of* f* (*W, H*) can be challenging due to the existence of local minima owing to the fact that *f* (*W*, *H*) is non-convex in both *W* and *H*. As noted before, due to its iterative nature, the NMF algorithm may not necessarily converge to a unique solution on every run. For a particular NMF solution of *W* and *H*, *WDD*^−1^* H* is is also a solution for any nonnegative invertible matrix *D *[42]. The NMF solution depends on the initial conditions for *W* and *H*. To address this issue, we use the Nonnegative Double Singular Value Decomposition (NNDSVD) initialization strategy proposed by Boutsidis and Gallopoulos [48]. This NNDSVD algorithm does not rely upon randomization and is based on approximations in the positive components of the truncated SVD factors of the original data matrix. Essentially, this provides NMF a fixed starting point, and the iteration to generate *W* and *H* will converge to the same minima. As noted by Chagoyen et al. in [36], having multiple NMF solutions does not necessarily mean that any of the solutions must be erroneous.

We use the multiplicative update algorithm proposed by Lee and Seung [49] to compute consecutive iterates of *W* and *H*:

	(4)

To avoid division by zero, the small constant 10^−9^ is added to the denominator of each update rule above. In each iteration, both *W* and *H* are updated, which generally gives faster convergence than updating each matrix factor independently of the other. The computational complexity of the multiplicative update algorithm is easily shown to be* O*(*kmn*) floating-point operations per iteration.

The choice of factorization rank* k* (selected number of features) is often problem-dependent, and it is a difficult problem to find the optimum value of* k.* In general,* k* is chosen such that it is less than the minimum dimension of *A* (*m* or *n*). We investigate the effect of rank* k* for classifying gene documents in the Results section. As discussed in [42,50], one can compensate for uncertainties in the data or to enforce desired structural properties in the factor matrices. Additional application-dependent constraints can be added by modifying the cost function of Equation (3) to be

where *α* and *β* are relatively small regularization (control) parameters and *J*_1_(*W*) and* J*_2_(*H*) are functions defining additional constraints (e.g., smoothness or sparsity) on *W* and *H*, respectively. As explained in [42,50], the rationale for enforcing smoothness  or sparsity constraints on the *W* factor is to potentially improve the interpretability of its feature (column) vectors. Applying such contraints to the columns of the *H* (coefficient) matrix factor can control the span (or use) of features to explain documents in the collection.

### FAUN workflow

**Figure 4:**
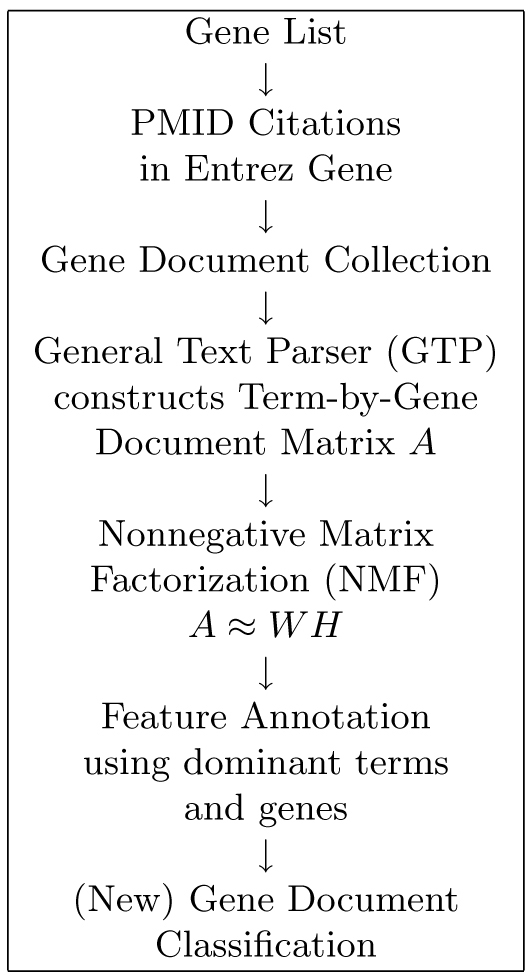
**FAUN workflow.** All genes in the gene list are used to construct a gene document collection from which a term-by-gene document matrix is constructed using GTP [46]. The matrix is then factored using rank *k* to produce a *k*-feature-NMF model. The resulting *W* and *H* matrix factors are used to extract dominant/significant terms and dominant genes for all *k* features. Dominant genes are then correlated for each feature. The FAUN user can annotate any feature and the resulting* annotated* NMF model can be used by the FAUN classifier to classify new gene documents.

As shown in workflow design depicted in Figure 4, for a given gene list, FAUN creates a* raw* term-by-gene document (sparse) matrix upon which an NMF model is built. The matrix for FAUN, created in the same manner as for SGO [32,33], is then decomposed using NMF methods. The raw matrix is factored using rank *k* to produce a *k*-feature-NMF model for the gene document collection. Currently, FAUN uses a rank *k* of 10, 15, and 20 for low, medium and high* resolution* of the NMF model, respectively. The NMF model containing *W* and *H* matrices is used to extract dominant terms and dominant genes for all *k* features. Dominant genes are then correlated for selected feature(s). FAUN users can then annotate some or all of the features and then deploy the FAUN classifier to determine the (concept-based) features of a new stream of gene documents.

The FAUN classifier accepts a new document to be classified, the entropy weights of terms in Equation (2) used in the NMF model, the term-by-feature matrix factor (*W*), stop words, and thresholds for entropy weight and term frequency.

The module then computes the weight for each feature based on the weight of its terms whose entropy is larger than the entropy threshold and frequency is larger than the term frequency threshold. It then outputs the features sorted by weight from the highest to the lowest. The process of mapping features to gene classes will be described below.

Preliminary testing indicated that the classifier accuracy was around 80%. The test was conducted based on the first dataset (described below) that contained 50 genes. NMF models were first built with ranks of 10, 20, 30 and 40 using 40 genes randomly selected from the 50-gene dataset. The classifier was then trained using the matrix in newly built NMF models. The accuracy was tested using the remaining 20% of the gene documents.

### Automated feature annotation

The FAUN classifier described above classifies genes based on annotated features in the NMF models. The process of annotating the features is typically done manually with the FAUN interface while exploring the gene dataset. Features in he NMF model can be annotated manually by the domain-expert using dominant feature terms. To automate the process for the other two datasets, features are annotated or mapped to gene classes using the FAUN annotation script (see [50]). In order to assign classfication categories (classes) to the genes, the script requires the matrix from the NMF model, the (known) classification categories, the NMF rank, and a feature weight threshold.

## Results

To evaluate the performance of FAUN in both classification and knowledge discovery tasks, we used three manually constructed gene document datasets with known functional gene relationships as a gold standard [51]. The first dataset (50TG collection) is a gene document collection of 50 manually selected genes related to development, Alzheimer’s disease, and cancer biology [33]. The second dataset (BGM collection) is composed of three non-overlapping gene lists from the Biocarta, Gene Ontology and MeSH databases [52]. The third dataset (NatRev collection) is composed of five gene lists selected from Nature Reviews papers [53-57]. Categories used in all of these datasets are shown in Table 1.

**Table 1:** List of categories for each dataset used to evaluate FAUN classification performance. GC is the gene count per category.

Dataset 1 (50TG)	
GC	Category
15	Cancer
11	Alzheimer
5	Development
16	Cancer & Development
3	Alzheimer & Development

**Dataset 2 (BGM)**	
**GC**	**Category**

21	Biocarta: Caspase cascade in apoptosis
8	Biocarta: Sonic hedgehog pathway
10	Biocarta: Adhesion and diapedesis of lymphocytes
10	GO: Biological process: telomere maintenance
7	GO: Cellular constituent: cornified cell envelope
20	GO: Molecular function: DNA helicase
8	MeSH: Disease: retinitis pigmentosa
8	MeSH: Disease: retinitis pancreatitis
10	MeSH: Disease: nephroblastoma (Wilm’s tumor)

**Dataset 3 (NatRev)**	
**GC**	**Category**

26	Autism
10	Diabetes
25	Translation
37	Mammary Gland Development
12	Fanconi Anemia

### Parameterization

Proper initialization of the NMF factors* W* and* H* is an important consideration for reproducibility (i.e., uniqueness of the factorization). The NNDSVD (see Methods for details) is one such approach for generating a robust and consistent factorization. NNDSVD basically starts with the truncated SVD of the gene-by-document (sparse) matrix* A.* Although NNDSVD produces a static starting point, different methods (see [43,50]) can be applied to remove zeros from the initial approximation and thereby prevent them from being fixed throughout the (multiplicative) update process.

The values of factorization ranks considered for the three datasets were 5, 10, 15, 20, 25, 30, 40, and 50. We restricted the maximum number of iterations to 1000 and 2000 and stopped iterations if the consecutive iterates of *W* and *H* (generated by the multiplicative update algorithm defined in Equation (4)) if the consecutive iterates of *W* and *H* were closer than *τ_W_* = 0.01 and* τ_H_* = 0.001, respectively, in Frobenius norm. That is, ||*W_old_* − *W_new_*||*F* <*τ_W_* and ||*H_old_* − *H_new_*||*F* <*τ_H_*. The effect of contraints on smoothing and sparsity for the *W* and *H* iterates has been studied and we refer the reader to [43,50] for more details on these effects.

### Classification accuracy

**Figure 5:**
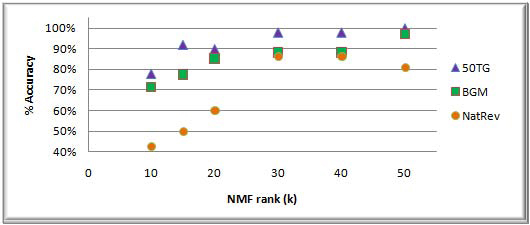
FAUN classification accuracy based on the strongest feature associated with each gene. [51]

For each dataset, six NMF models were generated with rank *k* set to 10, 15, 20, 30, 40 and 50. Features in the NMF models were annotated using the automated annotation process described in the Methods section. Using these annotated features, each gene in the dataset was assigned to the same category with its strongest annotated feature (indicated by the largest corresponding column entry of *H*). For example, if gene X has the strongest association with feature Y, then gene X will be classified as being in the category with which feature Y is labeled. The FAUN classification using the strongest feature (per gene) with rank *k* = 30 yielded 98%, 88.2% and 86.4% accuracy for datasets 1, 2, and 3, respectively (Figure 5).

The total gene recall per category was also investigated. For each feature, the corresponding maximum row entry of *H* (max *H*) is found and all genes in the feature with their *H* values < max *H* × *f_τ_*, where ƒ*_τ_* is a chosen feature threshold, are skipped. For each gene, all features (above *f_τ_*) associated with this gene are taken and then categories are assigned to the gene based on feature labeling.

The classification accuracy is evaluated in such a way that if the correct class is not among the classes assigned, the correctness is defined to be 0. If the correct class is among the classes assigned, the correctness is defined to be 1/(number of classes assigned to the gene). The total correctness is the sum of correctness assigned to every gene expressed as a percentage (0-100%). Using a feature weight threshold ƒ*_τ_* = 1.0, gene recall ranges of 78%-100%, 71.6%-97.1%, and 42.7%-80.9% for the 50TG, BGM, and NatRev datasets, respectively, have been reported [51].

Low classification accuracy equates to the misclassification of a human-curated category in the dataset. However, this misclassification does not necessarily imply that FAUN cannot be used to infer new (previously unknown) functional properties. A a few examples of such discovery are mentioned in the next section.

### Knowledge discovery

One of the most important capabilities of FAUN is to discover novel gene relationships from the biomedical literature, leading to generation of experimental hypotheses by the end user (biologist). This is essentially accomplished by clustering genes according to word usage patterns from the literature. By altering parameters in FAUN, a user can control the granularity by which genes and terms/features are associated. In this section we evaluate the effect of two parameters (rank-*k* and *H*-matrix threshold) on the discovery process using the NatRev dataset that contains 26 Autism associated genes from a total of 110 genes in the dataset.

**Figure 6:**
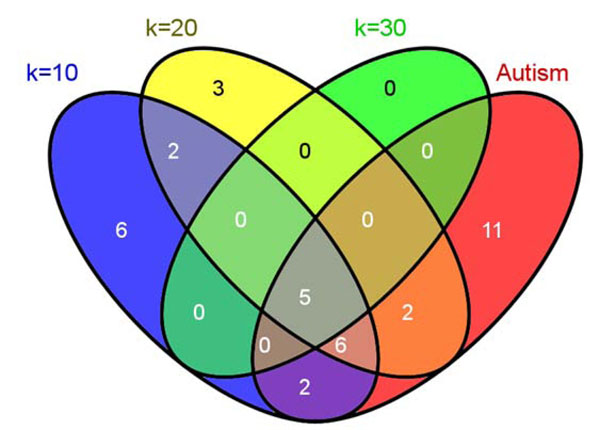
Venn diagram of genes from different NMF (rank-*k*) models generated from Autism gene documents in the NatREv collection.

**Figure 7:**
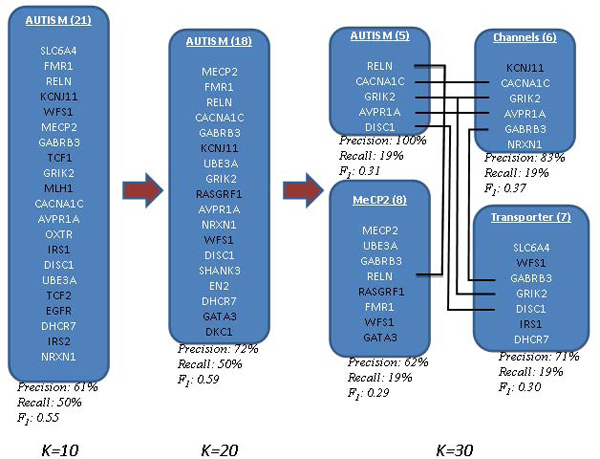
Gene distributions across different features from NMF (rank-*k*) models.

**Figure 8:**
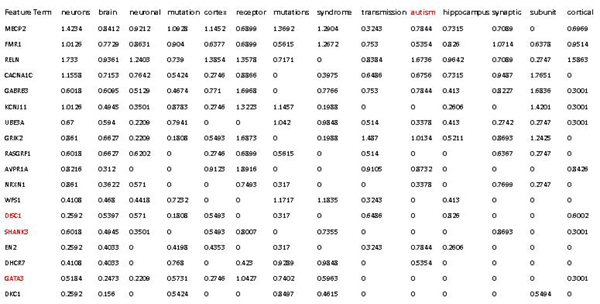
Matrix of genes by feature terms for the rank-20 NMF model of the NatRev collection.

Role of rank-*k* on discovery process. First, we examined gene clustering at various rank-*k*, using a constant global *H*-threshold. In general, the smaller the rank-*k* the larger the number of genes associated with each feature. For example, the feature containing autism as a highly ranked term (hereafter called* autism feature)* included 21 genes at* k* = 10 and only 5 genes at* k* = 30 (Figure 6). Interestingly, all 5 genes in the *k* = 30 autism feature are clearly linked to autism (100% precision) [53]. Using the gene list in the review article by Abrams and Geschwind as ground truth, precision, recall and F1 score (harmonic mean of precision and recall) values were calculated for the various features in this collection. Figure 7 shows that precision increases with *k* at the cost of recall and that rank-20 feature shows the best performance (highest F1 score). By examining the top ranked terms associated with this feature, we can see that the genes are clustered together because they share highly weighted terms such as neurons, brain, neuronal, mutation, cortex, receptor, syndrome, transmission, autism, hippocampus, synaptic and so on (Figure 8). Although useful, these terms appear to be somewhat general and do not adequately classify the genes into functional groups. For more specific annotation, a larger rank-*k* is required. For instance, at *k* = 30, the genes associated with autism by Abrams and Geschwind into 4 different features. These features are associated with more specific terms that better represent the gene functions, such as methylation, channels and transporters (Figure 7).

It is important to point out that NMF clusters genes together even if they do not share every top weighted term for the feature. For instance, the autism feature for the *k* = 20 model included **DISC1**, **SHANK3** and **GATA3** although the term autism did not appear in the abstracts used to build the NMF model (Figure 8, red highlighted genes and terms). Indeed, the abstracts used in our collection were limited to 2006 and earlier and the discovery of **SHANK3** and **DISC1** as autism genes occurred only after 2007 [58,59]. This association is due to the overlap of other terms that are highly weighted in this feature, demonstrating the utility of NMF for discovering new gene associations based on word pattern usage.

**Figure 9:**
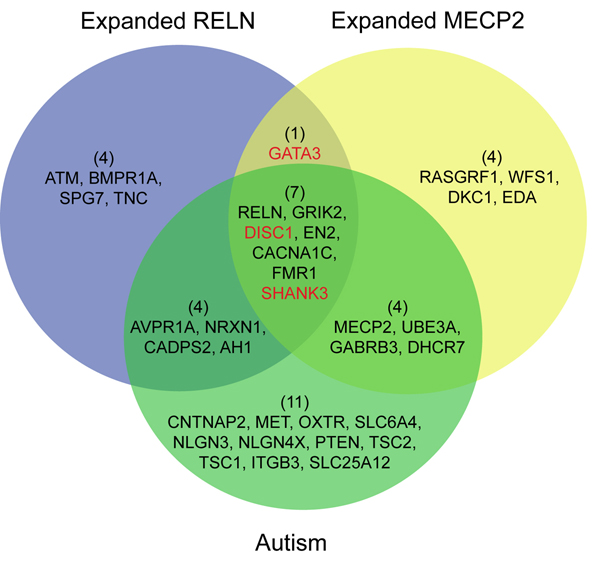
**Venn diagram of genes associated with Autism (RELN-related, blue) and methylation (MECP2-related, yellow) features using a lower threshold for *H*-matrix in the rank-30 NMF model of the NatRev collection.** The resulting genes sets were compared to the 26 autism associated genes reported by Abrams and Geschwind (green). Both the expanded RELN and expanded MECP2 gene sets achieved an F1 score of 0.52 (69% precision, 42% recall), whereas the union of the two gene sets achieved an F1 score of 0.63 (62% precision, 58% recall). Red highlighted genes were new discoveries identified by adjusting the rank-*k* on the same dataset (Figure 7).

Role of *H*-matrix threshold on discovery process. Another way to view more genes associated with a specific feature is to loosen the *H*-matrix threshold for that feature (set at a global median value of 1.0 by default). The threshold can be modified based on a local value. Lowering the local *H*-matrix threshold expands the number of genes associated with the feature. We applied this strategy to expand the number of genes associated with the autism and methylation features produced by the rank-30 NMF model in Figure 7 to a total of 16 genes each. Calculation of the F1 score using the Abrams and Geschwind dataset on autism, revealed that both sets achieved reasonable precision and recall (Figure 9). Interesting, the union of these two sets achieved a better F1 score than that achieved in *k* = 20 autism feature described above. Therefore, this strategy is capable of identifying additional target genes. Importantly, both genesets contained **GATA3**, which has not been definitively linked to autism to date.

Taken together, we presented two possible strategies here that can be used within FAUN to explore relationships between genes and to make predictions that can later be tested experimentally. Related genes may be identified by lowering the rank-*k* and using general terms to cluster genes together. Alternatively, related genes can be identified by lowering the *H*-matrix threshold on a higher rank-*k* model, which uses more specific terms to cluster genes together. Each strategy has its own merit and would likely produce different results with different datasets. It is important to point out that both strategies produced reasonably high precision and recall.

### Computational complexity

The cost per each NMF iteration typically ranges from 0.004 seconds to 0.011 seconds, depending on the choice of rank *k*, on a PC with 2 Intel Core CPU T5600/1.83 GHz processors, 1.5GB memory, running 32 bit Linux. The elapsed CPU time and operations per iteration of the NMF multiplicative update algorithm per dataset are listed in Table 2. Theoretically, one can estimate the complexity (floating-point operation count) of one iteration of Equation (4) to be *k* × *m* × *n*. This computational cost is compared with other competing NMF-update approaches in [50]. Overall, for the three datasets considered in this study, the runtimes are modest for a desktop/laptop computing environment.

**Table 2:** Computational cost of the NMF multiplicative-update algorithm.

NMF Rank *k*	Number of iterations			CPU Time (seconds)			Millions of ops. per iteration		
	
	50TG	BGM	NatRev	50TG	BGM	NatRev	50TG	BGM	NatRev
10	86	130	92	3.57	14.40	8.20	4	13	14
20	114	133	106	11.55	28.96	15.88	8	26	29
30	162	154	119	28.34	46.57	23.91	13	39	43
40	166	165	147	36.27	70.40	40.59	18	51	57
50	634	171	180	197.96	94.70	61.33	22	64	72

The number of terms (*m*) for the 50TG, BGM, and NatRev datasets are 8, 750, 12, 590, and 13, 038, respectively. The number of gene documents (*n*) for the 50TG, BGM, and NatRev datasets are 50, 102, and 110, respectively.

## Conclusions

In this study, we have developed a software environment called FAUN which implements nonnegative matrix factorization to extract gene associations from the biomedical literature. The tool was evaluated using three different gene sets as ground truth. Given a list of genes, FAUN allows researchers to not only hypothesize* why* genes might be related but also classify them functionally with promising accuracy. FAUN not only assists researchers to use biomedical literature efficiently, but also provides utilities for knowledge discovery which is particularly important for interpretation of discovery-oriented genomic data.

## Competing interests

The authors declare that they have no competing interests.

## Authors' contributions

This work is based on the PhD dissertation of Dr. Elina Tjioe, a former graduate student in the Graduate School of Genome Science and Techonology at the University of Tennessee. Under Professor Berry’s supervision, Dr. Tjioe designed and implemented the FAUN core modeling and interface software. Professor Homayouni provided gene lists and conducted extensive testing with the NMF models (i.e., features) generated by Dr. Tjioe. Professor Berry and Dr. Tjioe were primarily responsible for the Background and Methods and the screenshots and computational complexity components of the Results section. Professor Homayouni provided the knowledge discovery components of the Results section related to the NatRev (Autism) collection.

## Availability and requirements

Project name:	FAUN

Project home page:	https://grits.eecs.utk.edu/faun

Operating system:	Linux 2.6.18-128.1.10.el5

Programming language(s):	PHP 5.1.6 (cli), C++ (gcc version 4.1.2)

Source code restrictions:	License needed
